# Risk-Stratified Use of Topical and Infiltrative Local Anesthetics in High-Risk Dermatologic Surgery

**DOI:** 10.26502/jsr.10020489

**Published:** 2026-02-09

**Authors:** Seyedshayan Shojaei, Kimia Heidari, Alhasan Alobaidi, Devendra K Agrawal

**Affiliations:** 1 Department of Translational Research, College of Osteopathic Medicine of the Pacific, Western University of Health Sciences, Pomona, California 91766 USA; 2 School of Medicine, University of California at Irvine, Irvine, Irvine, CA 92697 USA

**Keywords:** Anesthetics, Local, Epinephrine, Dermatologic Surgical Procedures, Methemoglobinemia, Drug Toxicity

## Abstract

Local anesthetics are fundamental to dermatologic practice, yet their safety profile requires nuanced understanding in high-risk contexts including end-arterial sites, barrier-compromised skin, and scenarios predisposing to systemic toxicity. This narrative review synthesizes contemporary evidence across these three interacting domains to provide an integrated, risk-stratified framework for clinical decision-making. Regarding end-arterial territories, over two decades of clinical evidence encompassing more than 200,000 digital and acral injections has effectively dismantled the historical dogma against epinephrine use in digits, nose, ear, and penis, demonstrating an excellent safety profile when dilute concentrations are used in patients with adequate perfusion, with phentolamine providing reliable rescue for rare, prolonged vasoconstriction. In barrier-compromised skin (e.g. burns, ulcers, and inflammatory dermatoses) topical anesthetics function as absorption amplifiers, with dramatically accelerated systemic uptake that can precipitate local anesthetic systemic toxicity or prilocaine- and benzocaine-induced methemoglobinemia, particularly in infants and frail elderly patients. For large, denuded areas, dilute tumescent infiltration offers a pharmacokinetically safer alternative to high-dose topical therapy. The review details systemic toxicity risk factors, recognition, and management, emphasizing that intravenous lipid emulsion therapy has transformed severe toxicity from an often-fatal event to a manageable emergency. Special considerations for pediatric and geriatric populations, drug interactions, and cumulative dosing across modalities are addressed. The overarching conclusion is that context-sensitive risk stratification which includes integrating vascular status, barrier integrity, and host pharmacokinetics combined with office preparedness including phentolamine and lipid emulsion, enables safe local anesthesia even in traditionally high-risk dermatologic scenarios.

## 1. Introduction

### 1.1 Reframing Safety: Beyond Routine Administration

For the purposes of this review, we define “high-risk dermatologic contexts” as scenarios in which small deviations in agent selection, dose, or technique can disproportionately increase the probability or severity of ischemic injury, systemic toxicity, or methemoglobinemia. These contexts arise from three interacting domains: anatomy, barrier status, and systemic milieu. Anatomically, end-arterial or functionally end-arterial territories (digital pulps, nasal tip, ear, penis) have historically been considered precarious with respect to vasoconstrictors, particularly in patients with peripheral arterial disease, Raynaud phenomenon, or thromboangiitis obliterans [1–3]. From a barrier perspective, partial-thickness burns, chronic leg ulcers, erosive disorders, and acutely inflamed dermatoses amplify transcutaneous absorption of topicals and modify the pharmacokinetics of infiltrative agents [4,5]. Systemically, extremes of age, frailty, hepatic or cardiac impairment, and co-medications that inhibit cytochrome P450 enzymes or lower seizure threshold (e.g. β-blockers, SSRIs, TCAs, class I antiarrhythmics) further narrow the therapeutic window [6–8].

In such settings, global declarations of local anesthetic “safety” are insufficient. Instead, dermatologic practice requires a risk-stratified approach that integrates microvascular, barrier, and pharmacologic principles with the realities of office-based surgery: limited monitoring, variable emergency preparedness, and the increasing use of high-dose tumescent and topical regimens.

### 1.2 Objectives and Scope of This Review

This narrative review synthesizes and critically appraises the evidence base for the safety of topical and infiltrative local anesthetics in three interlocking high-risk domains central to dermatologic practice: end-arterial sites, barrier-compromised skin, and systemic toxicity. Our primary aim is to move beyond aphorism and isolated case reports toward an integrated framework that links drug structure, pharmacokinetics, vascular physiology, and clinical outcomes, and that can be translated into concrete, context-sensitive practice recommendations.

First, we examine the historical dogma and contemporary evidence for epinephrine safety in end-arterial territories. Second, we address the ‘absorption amplifier’ effect of barrier compromise, comparing topical anesthetic behavior across intact, burned, ulcerated, and inflamed skin. Third, we provide a comprehensive framework for LAST risk factors, recognition, and management.

The intended audience includes dermatologic surgeons, cutaneous oncologists, procedural and cosmetic dermatologists, pediatric dermatologists, hand and reconstructive surgeons using wide-awake local anesthesia, and allied specialties that intersect with dermatologic anesthesia. By integrating pharmacologic foundations (Section II), site-specific risk analyses (Sections III and IV), systemic toxicity frameworks (Section V), population-tailored considerations (Section VI), and procedural strategies (Section VII), we aim to provide a cohesive, practice-oriented synthesis.

The goal of this critical review article is to preserve the recognition that local anesthetics are extraordinarily safe, while making explicit the contextual limits of that safety.

### 1.3 Literature Search and Selection

This narrative review was informed by a targeted, non-systematic search of the biomedical literature, with PubMed as the primary database. We searched from database inception through late 2025 using combinations of MeSH terms and free-text keywords related to our three focal domains, including “local anesthetic,” “lidocaine,” “bupivacaine,” “prilocaine,” “topical anesthetic,” “tumescent anesthesia,” “epinephrine,” “digital nerve block,” “end-arterial,” “digits,” “nose,” “ear,” “penis,” “burn,” “ulcer,” “barrier-compromised skin,” “methemoglobinemia,” and “local anesthetic systemic toxicity” (LAST). Additional relevant terms (e.g. “WALANT,” “wide-awake local anesthesia,” “pediatric,” “geriatric,” “peripheral arterial disease,” “Raynaud,” “Buerger’s disease”) were added iteratively as themes emerged. We restricted inclusion to human studies and English-language publications and prioritized original clinical data which was then supplemented by high-quality narrative reviews, pharmacologic monographs (e.g. StatPearls), and key guideline or consensus pieces where available. Study selection and appraisal were pragmatic and purpose-driven rather than protocolized. No formal risk-of-bias assessment or meta-analysis was performed.

## 2. Pharmacologic foundations relevant to high-risk contexts

Local anesthetic safety in end-arterial sites, barrier-compromised skin, and large-field tumescent applications is fundamentally determined by the interplay between drug structure, pharmacokinetics, and the modifying effects of vasoconstrictors. Understanding these pharmacologic foundations is essential to rational risk assessment in settings where a small margin separates effective regional anesthesia from local anesthetic systemic toxicity (LAST), ischemia, or methemoglobinemia.

### 2.1 Pharmacokinetic Determinants of Safety

Lipid solubility and protein binding are the primary determinants of local anesthetic potency and duration. However, volume of distribution and elimination half-life further modulate risk. Lidocaine exhibits a relatively large volume of distribution between 0.7 and 1.5 L/kg and an elimination half-life between 1.5 and 2.0 hours in healthy adults, so transient overshoot in plasma levels is usually rapidly corrected if further absorption is curtailed [9,10]. Bupivacaine has a high degree of plasma protein binding (about 95%) and a longer elimination half-life than lidocaine: adult studies report a half-life of 2.7 hours, within a broader published range from 1.5 to 5.5 hours, so it accumulates more readily with repeated dosing or impaired clearance, and its high affinity for cardiac sodium channels means that malignant ventricular arrhythmias and cardiovascular collapse may occur at plasma concentrations only slightly above the therapeutic range [9,11,12]. Prilocaine is intermediate in half-life but carries a qualitatively distinct risk: metabolism to o-toluidine oxidizes hemoglobin to methemoglobin, producing cyanosis and tissue hypoxia at plasma levels that may still be sub-toxic for CNS and cardiovascular systems [13,14].

These properties translate into agent-specific toxicity profiles that are particularly relevant in high-risk dermatologic contexts. Lidocaine, because of its moderate lipid solubility and relatively low cardiotoxicity, typically manifests CNS excitation (tinnitus, peri-oral numbness, seizures) before cardiovascular collapse when LAST occurs, allowing a broader window for recognition and intervention [15]. Bupivacaine and, to a lesser extent, ropivacaine may precipitate abrupt ventricular arrhythmias or asystole with only minimal antecedent neurologic warning, making them less forgiving in office-based settings with limited resuscitative capacity [9]. Prilocaine, particularly in topical eutectic mixtures of local anesthetics (EMLA; 2.5% lidocaine / 2.5% prilocaine), can elicit clinically significant methemoglobinemia in infants, patients with G6PD deficiency, and when applied to large areas of barrier-defective skin [13,14].

Thus, the choice of agent in digits, acral sites, or on denuded dermis should be informed by more than duration alone. Highly lipophilic, long-acting agents (bupivacaine) should be reserved for limited-volume nerve blocks in monitored settings, whereas lidocaine (with or without prilocaine in topical mixtures) should be used with strict surface area and dose limits when applied to barrier-compromised skin [9].

### 2.2 The Epinephrine Paradox: Adjuvant and Risk Modifier

Epinephrine (adrenaline) is the prototypical vasoconstrictor adjuvant in local anesthetic solutions. By activating α₁-adrenergic receptors on arteriolar smooth muscle, it reduces local blood flow, thereby decreasing systemic uptake, prolonging nerve block duration, and improving intraoperative hemostasis [16,17]. These absorption-flattening effects are central to the safety of high-volume tumescent anesthesia and allow larger total lidocaine doses to be used with acceptably low peak plasma levels (section 5.1).

Epinephrine’s longstanding avoidance in end-arterial sites is revisited in detail in section III. This creates the central paradox: its α₁-mediated vasoconstriction is the primary safeguard against systemic toxicity in high-volume anesthesia (by slowing uptake and lowering C_max), yet this same mechanism is the source of the historical fear regarding ischemic necrosis in digits.

## 3. End-arterial sites: dismantling dogma with evidence

Local anesthetic use in end-arterial territories which includes digits, nasal tip, ear, and penis has historically been constrained by the fear that epinephrine-induced vasoconstriction could irreversibly occlude already tenuous blood flow. Contemporary pharmacologic and clinical data, however, demonstrate that this fear is largely unfounded when dilute epinephrine is used with modern amide anesthetics and meticulous technique.

### 3.1 Contemporary Evidence for Safety in Digits and Acral Sites

Over the past two decades, a robust body of evidence has emerged refuting the notion that dilute epinephrine in digital anesthesia causes ischemic necrosis in otherwise viable fingers or toes. A 2015 systematic review by Ilicki identified 23 studies encompassing 2,797 digital nerve blocks performed with lidocaine–epinephrine at concentrations between 1:100,000 and 1:200,000; no epinephrine-related cases of irreversible digital ischemia were identified [27]. In the Dalhousie multicenter prospective study, Lalonde and colleagues reported 3,110 consecutive elective finger and hand procedures performed with lidocaine and epinephrine, without any instances of digital infarction or need for amputation [28]. A separate prospective cohort of 1,340 digital surgeries using lidocaine 1% with epinephrine 1:100,000 likewise reported no ischemic complications or tissue necrosis [2].

Large prospective and retrospective cohorts corroborate this safety profile. Beyond the Dalhousie series, subsequent WALANT reports have described several hundred to many thousands of hand procedures performed with lidocaine and epinephrine, again without epinephrine-attributed digital tissue loss [2]. When these modern hand-surgery data are considered together with the classic podiatric series of more than 200,000 forefoot and toe operations performed using lidocaine with epinephrine at concentrations of 1:100,000 to 1:200,000, the published literature now documents well over 200,000 acral injection (including more than 200,000 from podiatric series alone plus several thousand from hand-surgery cohorts) without a single confirmed case of digital infarction attributable to epinephrine [28].

Similar safety profiles extend to other acral sites. Häfner and colleagues reported more than 10,000 ear and nasal procedures performed with epinephrine-supplemented local anesthetics at concentrations in the range of 1:100,000 to 1:200,000, without flap loss or skin necrosis attributable to vasoconstriction [29]. In a subset of these patients, perfusion measurements at the earlobe showed a 69% reduction in laser Doppler blood-flow signal and a 42% reduction in arterial inflow immediately after injection, yet blood supply remained present and no tissue necrosis occurred [29].

Physiologic studies provide mechanistic support. In a double-blind randomized trial of 20 healthy volunteers, Häfner et al. found that digital Oberst blocks using 6 mL of lidocaine 1% with epinephrine 1:200,000 reduced acral blood flux by a maximum of 55% for a mean duration of 16 minutes; perfusion measurements at 6 hours and 24 hours were indistinguishable from baseline [29]. In a separate WALANT study of 17 patients, Moog et al. injected 5 to 7 mL of articaine 1% with epinephrine 1:200,000 at the finger base and observed at least a 30% drop in capillary-venous oxygen saturation in 7 patients and short episodes of critical oxygen saturation in 4 patients, each lasting a mean of 133 seconds; oxygen saturation had returned to non-critical values in all patients by the end of the 32-minute observation period and no postoperative ischemic complications were seen [30]. Taken together, these data indicate that standard clinical doses cause a marked but short-lived reduction in digital perfusion that normal tissues tolerate without infarction.

Even in extreme “stress tests” of digital circulation such as accidental auto-injector injuries with epinephrine 1:1,000 into a single finger, permanent tissue loss has been exceedingly rare. Fitzcharles-Bowe et al. reviewed 59 reported cases of high-dose epinephrine injection into digits and found no instances of digital necrosis, including 32 patients who received no specific vasodilator treatment [3]. In a separate poison-center cohort, Muck et al. identified 365 epinephrine injections to the hand over six years; 213 involved digits and 127 of these digital injections had documented follow-up. Four patients had transient ischemic changes, all of which resolved completely, and in two of these patients symptoms resolved within 2 hours; no patient required hospitalization, hand-surgery consultation, or surgical intervention [2]. These observations imply a substantial safety margin for dilute epinephrine in digital blocks, which use much smaller epinephrine doses than auto-injectors.

Collectively, contemporary clinical and physiologic evidence demonstrates that, in healthy digits and in most patients with common comorbidities, lidocaine with epinephrine at 1:100,000–1:200,000 provides longer anesthesia and superior hemostasis without a demonstrable increase in the risk of digital necrosis [2,3].

### 3.2 Risk Stratification in Compromised Vasculature

#### 3.2.1 Peripheral Arterial Disease and Diabetes:

The reassuring safety data for epinephrine-containing anesthetic in end-arterial sites largely derive from populations with normal or only mildly impaired digital perfusion. Nonetheless, limited evidence suggests that even patients with common vascular comorbidities tolerate epinephrine well when perfusion is clinically adequate. Several WALANT cohorts have explicitly included substantial proportions of patients with hypertension, diabetes, smoking history, or antiplatelet and anticoagulant therapy, yet none of these series reported digital ischemia, blistering, or necrosis attributable to epinephrine [2]. Other reports have deliberately included patients described clinically as having “poor circulation” and still found no epinephrine-related necrotic complications [2].

Notably, most large WALANT series either excluded patients with overt ischemic signs (rest pain, tissue loss, prior digital infarction) or used epinephrine cautiously or not at all in those with critical limb ischemia, Buerger’s disease, or severe scleroderma [28]. Thus, the observed absence of necrosis in “at-risk” circulation likely reflects a combination of true safety in mild–moderate peripheral arterial disease (PAD) and selection bias away from those with severely compromised flow.

From a pragmatic standpoint, the absence of documented epinephrine-related necrosis in diabetics and patients with non-critical PAD suggests that routine exclusion of epinephrine in all such patients is unnecessarily conservative. However, when objective measures such as ankle–brachial index (ABI <0.4), monophasic toe pressures, tissue loss, or prior digital amputations indicate severely impaired perfusion, the marginal benefit of epinephrine (longer anesthesia, better hemostasis) may be outweighed by the theoretical risk of tipping precarious microcirculation into infarction. In these individuals, plain lidocaine or proximal nerve blocks without epinephrine remain reasonable alternatives.

#### 3.2.2 Vasospastic Disorders (Raynaud phenomenon, Buerger’s Disease):

Patients with primary Raynaud phenomenon, secondary Raynaud’s due to connective tissue disease, or thromboangiitis obliterans (Buerger’s disease) represent a distinct category in whom α-adrenergic vasoconstriction may elicit exaggerated and prolonged digital vasospasm. Case reports describe unusually severe ischemic responses: for example, a patient with Raynaud’s who developed marked digital pallor, pain, and superficial blistering after a standard epinephrine-containing injection, with eventual but delayed reperfusion [1]. Historical cases of digital necrosis in scleroderma or mixed connective tissue disease have also been reported, though confounding factors such as infection and baseline microvascular obliteration complicate causal attribution [2].

Pathophysiologically, Raynaud’s digits exhibit hypersensitivity of α_2-adrenergic receptors on digital arteries and arterioles, leading to disproportionate vasoconstriction in response to cold or catecholamines; Buerger’s disease is characterized by segmental inflammatory thrombosis of small and medium arteries and veins. Superimposing pharmacologic vasoconstriction on such structurally or functionally compromised vessels could, in theory, produce critical ischemia even with doses safe in normal digits. This theoretical vulnerability, coupled with case-level signals, has led most WALANT proponents to list active severe Raynaud’s and Buerger’s disease among the few relative contraindications to epinephrine in digital blocks [28].

In the absence of robust prospective data, a conservative posture remains prudent: avoid epinephrine in patients with clinically evident vasospastic episodes, rest pain, or trophic changes, and favor plain lidocaine or more proximal blocks in these individuals.

#### 3.2.3 Proposed Risk Assessment Framework:

Given the heterogeneity of vascular reserve among patients, a binary “epi or no epi” rule is inadequate. Instead, a structured risk assessment may be more appropriate. Clinically relevant elements include patient-level factors such as documented PAD (ABI and toe pressures), diabetes duration and complications, smoking history, prior digital ulcers or amputations, Raynaud’s attacks, and systemic vasculitis, as well as procedure-level factors such as the planned anatomic site, depth of dissection, anticipated bleeding, and the total volume and concentration of epinephrine.

A practical framework might categorize patients into low, intermediate, and high vascular risk. Low-risk patients have normal pulses, no history of ischemic events, and no systemic vasculopathy; in them, standard concentrations of lidocaine with epinephrine (1:100,000–1:200,000) can be used freely in digits, nose, ear, and penis, with phentolamine available for rare prolonged blanching. Intermediate-risk patients include diabetics with intact but diminished pulses or smokers with mild PAD but no rest pain or tissue loss; in this group, epinephrine use remains reasonable but should be limited to the minimum effective concentration and volume, with careful monitoring of digital coloration and capillary refill post-injection and a low threshold for phentolamine reversal if reperfusion is delayed. High-risk patients such as those with critical limb ischemia (typically ABI ≤0.4 and/or toe pressures <30 mmHg), active ulceration or gangrene, prior digital infarction, severe vasospastic disorders, or inflammatory vasculitis, should generally avoid epinephrine in end-arterial injections; anesthesia should instead be achieved with plain lidocaine, proximal nerve blocks away from critically ischemic segments, or regional techniques under monitored conditions.

Such a qualitative algorithm, while not yet prospectively validated, concretizes the logic already applied in expert WALANT series, which systematically excluded patients with “significant pre-existing hand or finger ischemia” from epinephrine use [28]. Future work incorporating objective vascular measurements (ABI, toe pressures, nailfold capillaroscopy) and prospective outcomes could refine this into a validated digital perfusion risk score.

### 3.3 Phentolamine Rescue: The Safety Net

Phentolamine, a non-selective α-adrenergic antagonist, provides an effective pharmacologic antidote to epinephrine-induced vasoconstriction. By competitively displacing epinephrine at α₁-receptors, it induces rapid arteriolar dilation and restoration of blood flow [28]. In the context of digital anesthesia, phentolamine rescue should be considered whenever blanching, pain, or impaired capillary refill persists beyond the expected window of epinephrine effect, or at any earlier point if there are clinical signs of progressive digital ischemia, particularly in high-risk patients. Key clinical and physiologic data supporting the safety of epinephrine in end-arterial sites are summarized in table 2.

A commonly recommended protocol involves reconstituting phentolamine to 1 mg/mL and infiltrating 1–5 mg subcutaneously in and around the ischemic area, using multiple small injections circumferentially proximal to and within the original anesthetic field [28]. In an experimental human study, Nodwell and Lalonde showed that injecting 1 mg of phentolamine in 1 mL of saline at the site of vasoconstriction shortened the time for epinephrine-induced digital blanching to resolve from a mean of 5 hours 19 minutes with placebo to 1 hour 25 minutes with phentolamine [3]. Case reports of accidental high-concentration epinephrine auto-injector injuries describe rapid restoration of digital perfusion after local phentolamine injection, with preservation of tissue and no subsequent necrosis in the reported cases [2,3].

Timing is paramount. Experimental and clinical data indicate that digital tissues can tolerate only a limited period of severe ischemia before the risk of irreversible damage rises [31]. In a recent report of digital ischemia after an adrenaline-based block, delayed recognition and late administration of phentolamine were followed by only partial recovery of perfusion and distal tissue loss, suggesting that an earlier intervention might have prevented necrosis [32]. On this basis, several authors advocate administering phentolamine once it is clear that perfusion is not beginning to recover such as when normal coloration and capillary refill have not started to improve by 60 minutes in a previously healthy digit or sooner in patients with compromised vascular reserve.

Phentolamine itself is hemodynamically active; systemic absorption can cause transient hypotension and tachycardia, though these are usually mild at doses used for digital rescue [28]. Having phentolamine stocked and staff trained in its use therefore substantially enhances the safety net for using epinephrine in end-arterial sites. It converts a theoretical one-way door of vasoconstriction into a reversible pharmacologic state, further supporting the argument that with appropriate infrastructure, epinephrine in digits and other acral sites is not only safe but controllable.

## 4. Barrier-Compromised Skin: The Absorption Amplifier

### 4.1 Clinical Consequences of Excessive Absorption

#### 4.1.1 Systemic Local Anesthetic Toxicity (CNS/Cardiovascular):

The amplified absorption from barrier-compromised skin translates into an increased risk of frank local anesthetic systemic toxicity (LAST). Case reports span pediatric and adult populations and frequently involve either diseased skin, extensive application, occlusion, or high-concentration compounded formulations. In a four-year-old child with atopic dermatitis and molluscum contagiosum, EMLA was applied under occlusion to numerous lesions; within a short period the child developed seizures and cyanosis, with documented methemoglobinemia and clinical features consistent with combined prilocaine-induced oxidant stress and systemic lidocaine toxicity [34]. A recent adult case involved a 71-year-old man with a chronic venous leg ulcer who received EMLA over the ulcer bed; within 45 minutes he became somnolent and cyanotic, with a methemoglobin level of 15.1% and central nervous system depression that resolved only after removal of the cream and supportive oxygen therapy [13].

Topical overuse on procedurally ablated skin can be equally hazardous. A report of fractional laser resurfacing described systemic lidocaine toxicity after the application of a 30% lidocaine gel to the treated area, with ensuing neurologic symptoms requiring emergent care [35]. The most dramatic illustration of this risk came in 2005, when two young women died after using high-strength, compounded lidocaine/tetracaine gels on their legs under plastic occlusion before laser hair removal [6,25]. Both developed seizures and cardiac arrest en route to treatment facilities. The subsequent FDA advisory explicitly linked the fatalities to the combination of large surface area, high concentration, barrier disruption from shaving, and occlusion, all of which accelerated systemic uptake [25].

These cases underscore that the canonical CNS prodrome of LAST (peri-oral numbness, tinnitus, lightheadedness, confusion) may be brief or even unrecognized when high systemic levels are achieved rapidly from compromised skin, particularly outside monitored settings [6,9]. For dermatologic practice, they mandate that topical regimens on barrier-compromised skin be considered pharmacologically equivalent to substantial systemic dosing and be prescribed and monitored with the same vigilance as infiltrative anesthesia.

#### 4.1.2 Methemoglobinemia

On compromised skin, rapid systemic delivery of prilocaine and benzocaine increases the risk of methemoglobinemia, particularly in infants and oxidant-vulnerable adults. Key risk factors in barrier-compromised contexts include high total dose, large surface area of denuded skin, mucosal application, and host vulnerability (age <6 months, G6PD deficiency, concurrent oxidant medications). Full pathophysiology, recognition, and management with methylene blue are detailed in section 5.3 [34,36].

### 4.2 Evidence-Based Application Algorithms by Disease State

Translating pharmacokinetic and toxicologic data into practical guidance requires disease-specific algorithms that explicitly adjust for barrier status.

In atopic dermatitis, the combination of increased permeability and inflamed microvasculature dictates conservative topical regimens. Data published by Juhlin demonstrate that on eczematous skin, EMLA achieves adequate anesthesia with contact times as short as 5–15 minutes [37]. For adults and older children with localized lesions, a thin layer of EMLA or 4–5% lidocaine cream limited to the minimal necessary area and removed after 15–30 minutes is usually sufficient, obviating the standard 60-minute exposure used on intact skin [9,37]. Occlusion should be avoided whenever the epidermis is visibly inflamed or fissured. In young children, particularly those under three years, lidocaine-only preparations in small quantities are preferable to prilocaine-containing EMLA to mitigate methemoglobin risk [13,14].

For chronic venous or pressure ulcers, the leg-ulcer pharmacokinetic studies define relatively generous but still safe limits within the PK range shown in table 3 [5,38]. In frail elderly or patients with significant hepatic impairment, lower doses with longer intervals are prudent.

Partial-thickness burns require the most caution. For analgesia during burn dressing changes, topical anesthetics should be restricted to discrete, limited areas and contact times kept to 30–45 minutes. When larger segments require debridement or grafting, staged procedures and dilute tumescent or regional infiltration offer a safer profile than attempting to anesthetize the entire field with topical agents [39,40].

For inflammatory dermatoses overall (psoriasis, lichen planus, erosive disorders), a pragmatic rule is to limit both dose per unit area and contact time to no more than half of the amounts used in intact-skin protocols, avoid occlusion over any visibly eroded surface, and favor lidocaine-only formulations in children, patients with G6PD deficiency, or those requiring repeated treatments [9,13,14].

### 4.3 Age-Stratified Safety Considerations

Age modifies the impact of barrier compromise on systemic exposure: infants/young children, and frail older adults, have much narrower therapeutic windows. Infants have a higher surface-area-to-body-weight ratio, immature hepatic metabolism, and reduced methemoglobin-reducing capacity [6,13,14]. Frail older adults often have reduced hepatic blood flow and polypharmacy that slows clearance.

In practice, this means that pediatric and geriatric dosing on barrier-compromised skin should be substantially more conservative than in healthy adults. Detailed age-specific recommendations including tight labeled pediatric limits and dose reductions in frail elders are provided in section VI and tables 3 and 4.

### 4.4 Topical vs. Tumescent Infiltration: Optimal Strategy for Large Areas

For large areas of barrier-compromised skin such as extensive partial-thickness burns, large ulcers, or wide erosive fields, the clinician must decide between escalating topical therapy and transitioning to dilute infiltrative techniques. Pharmacokinetic principles strongly favor tumescent or field infiltration for such indications.

Tumescent anesthesia employs very dilute lidocaine (typically 0.05–0.1%) with epinephrine, infiltrated in substantial volumes into subcutaneous tissue until tumescence is achieved [17]. The combination of extreme dilution and epinephrine-mediated vasoconstriction produces slow, delayed systemic uptake: in tumescent liposuction with a mean lidocaine dose of 33.2 mg/kg, mean peak serum lidocaine concentration is 2.3 μg/mL (standard deviation 0.63 μg/mL), occurring 5–17 hours after infiltration, and all observed values remain below 6 μg/mL, the commonly used threshold for mild systemic toxicity [26,41]. In burn surgery, analogous tumescent protocols using 0.1% lidocaine with epinephrine for debridement and grafting of extensive burned areas have been reported as simple, effective, and safe, with no clinically significant systemic toxicity and excellent analgesia and hemostasis [39,40].

In contrast, applying even moderate-concentration topical anesthetic over a large, denuded surface creates a rapid, uncontrolled absorptive interface. As the burn-ointment case illustrates, 5% lidocaine on 28% body surface area can produce near-toxic peaks within hours [4], whereas an equivalent or higher total dose delivered tumescently yields a much flatter concentration–time curve with a lower C_max and wider safety margin [17,26,41].

From a practical standpoint, topical anesthesia is best reserved for small, discrete compromised areas (for example ≤25–50 cm^2^) and as an adjunct to reduce injection pain. For large contiguous areas of partial-thickness injury or ulceration for example, treatment fields larger than 100 cm^2^, which far exceeds the maximum labeled intact-skin area of 20 cm^2^ for 2 g of EMLA in infants 3–12 months or when multiple sessions on the same field are planned, staged dilute infiltration or tumescent anesthesia is generally a safer and more controllable strategy than escalating topical doses. Epinephrine in the tumescent solution not only slows systemic lidocaine uptake but also provides superior hemostasis, which is particularly advantageous in debridement and grafting of vascular wound beds [17,42].

A rational decision framework thus weighs surface area, depth of injury, need for hemostasis, patient comorbidities, and the cumulative anesthetic burden. Small islands of erosive disease or isolated ulcers can be managed with carefully dosed topical agents. Larger, contiguous areas of barrier loss, particularly in adults with reasonable cardiopulmonary reserve, are better served by dilute lidocaine with epinephrine delivered via tumescent or field infiltration, with dose calculations anchored to tumescent safety data and the availability of monitoring and lipid rescue for rare systemic events [17,26,41].

## 5. Local Anesthetic Systemic Toxicity (Last): From Rare To Manageable

### 5.1 Risk Factors for Systemic Toxicity

#### 5.1.1 Dose and Concentration Variables:

Systemic toxicity is fundamentally dose-dependent, modulated by concentration, route, and rate of administration. Expert consensus and anesthesia literature provide maximum recommended mg/kg doses for infiltrative use of common agents (Table 5). For dermatologic practice, the key principle is that these limits represent upper bounds under ideal conditions; in frail, pediatric, or comorbid patients, and when multiple modalities (topical, infiltrative, tumescent) are combined, substantially lower thresholds are appropriate.

Concentration strongly influences both injection pain and systemic risk. Higher concentrations (e.g. 2% lidocaine) provide no additional depth of block over 0.5–1% for most cutaneous procedures but increase the per-milliliter drug load. Recent dermatologic trials show that 0.25–0.5% lidocaine with epinephrine provides non-inferior analgesia for Mohs surgery and excisions compared with 1–2% solutions, while 1:2 and 1:6 dilutions of 2% lidocaine with epinephrine reduce the per-milliliter lidocaine content by 66.7% and 85.7%, respectively, and significantly lower injection pain scores [49,50]. Using the lowest effective concentration is therefore a straightforward, evidence-based strategy to widen the safety margin.

As shown in pharmacokinetic work on tumescent anesthesia (section 7.1), maximum safe dose is tightly linked to concentration and absorption rate.

#### 5.1.2 Site of Administration:

Anatomic site influences systemic uptake through differences in vascularity and barrier integrity. Highly perfused areas such as the face, scalp, genitalia, and mucous membranes absorb local anesthetics more rapidly than the trunk or extremity skin, increasing peak plasma concentrations for a given dose [9]. Topical anesthetics on mucosa or denuded dermis behave pharmacokinetically more like parenteral administration [25].

By contrast, subcutaneous or intradermal infiltration into intact skin yields comparatively slow absorption, especially when epinephrine is included [16]. As detailed in Section IV, barrier-compromised skin can convert percutaneous absorption from a diffusion-limited to a perfusion-limited process, markedly increasing systemic exposure to topical agents.

#### 5.1.3 Patient-Specific Vulnerabilities:

Host factors can narrow the margin between therapeutic and toxic plasma levels. Extremes of age are particularly important. Neonates and young infants have immature hepatic cytochrome systems, reduced α-1-acid glycoprotein levels, and a higher unbound fraction of amide anesthetics, lowering the threshold for CNS and cardiovascular toxicity [13,14]. Their higher surface-area-to-weight ratio also amplifies systemic uptake from topicals [6].

Elderly and frail patients often have reduced cardiac output, diminished hepatic blood flow, and polypharmacy, all of which may slow anesthetic clearance and reduce physiologic reserve in the face of hypotension or arrhythmias [6]. In such individuals, conservative dosing (for example, a 20% reduction from standard adult maximum doses), use of dilute solutions, and avoidance of rapid, large boluses are prudent [6].

Hepatic dysfunction directly impairs metabolism of amide anesthetics (lidocaine, bupivacaine, mepivacaine, prilocaine), prolonging half-life and increasing AUC at any given dose [6]. Severe cardiac disease reduces hepatic perfusion and therefore clearance; concomitant heart failure also diminishes tolerance for negative inotropy or arrhythmias. Patients with advanced renal disease are less susceptible to unchanged drug accumulation but may accumulate active metabolites (e.g. prilocaine’s o-toluidine), heightening the risk of methemoglobinemia [13,14].

Finally, patients with pre-existing neurologic disease, seizure disorders, or medications that lower seizure threshold (e.g. SSRIs, TCAs) may manifest CNS toxicity at lower plasma concentrations [9,26,41].

### 5.2 The Cumulative Dose Problem

In contemporary dermatologic practice, patients increasingly undergo multiple procedures, including ablative laser resurfacing, serial photodynamic treatments, staged excisions, and combined aesthetic interventions, within compressed timeframes. Each encounter may involve topical anesthetics, infiltrative lidocaine, nerve blocks, or tumescent solutions. Although any single exposure may remain comfortably within recommended limits, cumulative doses across modalities and sessions can approach or exceed thresholds for toxicity if not consciously tracked.

Lidocaine has a plasma elimination half-life of 90–120 minutes in healthy adults, but with tumescent infiltration, peak serum lidocaine concentrations typically occur 8–14 hours after injection, within a reported range of 5–17 hours and clinically significant absorption and analgesia can persist for up to 18 hours [26,51]. Consequently, lidocaine administered in the morning may still contribute appreciably to plasma levels during an afternoon or evening procedure, particularly in patients with reduced clearance. Similar principles apply to prilocaine, where repeated topical applications in close succession can allow accumulation of oxidizing metabolites and progressive methemoglobinemia [13,14].

Case analyses from tumescent liposuction have shown that patients receiving cumulative doses near the upper recommended range may develop mild neurologic symptoms which includes drowsiness, confusion, perioral numbness at peak levels many hours post-procedure, especially when concomitant medications impair metabolism [26,41]. Although these events generally remain subclinical and self-limited, they underscore the potential for delayed toxicity when multiple large-dose exposures are temporally clustered.

Busy practices should therefore adopt explicit policies that treat all local anesthetic administered within a 24-hour interval as a single cumulative dose for safety calculations [26,41,51]. Electronic medical record prompts or dedicated dosing sheets can facilitate real-time summation of infiltrative, topical, and tumescent lidocaine (and other amide anesthetics), ensuring that the aggregate milligram load per kilogram remains within context-appropriate limits. Where same-day multiple procedures are unavoidable, clinicians should favor more dilute solutions, restrict topical surface area, and avoid stacking high-dose modalities (for example, combining high-dose EMLA on compromised skin with high-dose tumescent lidocaine).

### 5.3 Methemoglobinemia: A Distinct Toxicity Pathway

Methemoglobinemia represents a mechanistically distinct form of toxicity in which oxidizing local anesthetic metabolites convert ferrous (Fe^2+^) hemoglobin to the ferric (Fe^3+^) state, forming methemoglobin (MetHb) and impairing oxygen carriage and release [52]. Prilocaine and benzocaine are the principal culprits in dermatologic practice; lidocaine and articaine can contribute at high doses or in susceptible hosts but are far less potent oxidants [13,14].

Prilocaine’s metabolite o-toluidine is a well-recognized inducer of methemoglobinemia, particularly when cumulative doses exceed 2–2.5 mg/kg in infants or 600 mg in adults [44]. As a component of EMLA (2.5% prilocaine + 2.5% lidocaine), prilocaine has been implicated in cases of significant MetHb formation when applied to extensive eczematous skin, chronic ulcers, or under occlusion, especially in young children [13,34]. Benzocaine, widely used as a topical mucosal spray, has caused fulminant methemoglobinemia in infants and adults after relatively small exposures, reflecting its high oxidative potency and rapid mucosal absorption [36].

Risk factors include high total dose, young age (particularly <6 months, when NADH-methemoglobin reductase is immature), glucose-6-phosphate dehydrogenase (G6PD) deficiency, concurrent oxidant drugs (e.g. dapsone, nitrates, sulfonamides), anemia, and application to highly vascular or barrier-deficient surfaces [36,52]. In a series analyzing prilocaine-induced methemoglobinemia, higher prilocaine dose and younger age were the most significant predictors of elevated MetHb levels [53].

Clinically, patients present with slate-gray or cyanotic discoloration, disproportionate to measured arterial oxygen tension; pulse oximetry often plateaus around 80–85% despite supplemental oxygen, while PaO₂ remains normal [52]. Symptoms range from mild dyspnea and headache to confusion, tachycardia, and, at MetHb levels exceeding 30–40%, seizures, arrhythmias, and cardiovascular collapse may occur (methylene blue is typically indicated at MetHb ≥20% or with significant symptoms at lower levels).

Diagnosis is confirmed by co-oximetry, which directly quantifies methemoglobin fraction. Management hinges on immediate removal of the offending agent and, in symptomatic patients or those with MetHb ≥20%, administration of intravenous methylene blue at 1–2 mg/kg over 5 minutes (up to a total of 7 mg/kg), which accelerates reduction of methemoglobin via the NADPH-dependent pathway [13,14]. In G6PD deficiency, methylene blue may be ineffective or even harmful; in such cases, exchange transfusion or hyperbaric oxygen may be required [52]. Prevention in dermatologic practice rests on dose limitation of prilocaine-containing and benzocaine products, strict adherence to age-specific guidelines, and avoidance in known G6PD-deficient or very young infants [13,14].

### 5.4 Prevention Strategies

Robust prevention remains the most effective intervention against LAST and related toxicities. The cornerstones are accurate dose calculation, prudent technique, judicious use of vasoconstrictors, and appropriate monitoring.

Weight-based dosing should be routine for children, small adults, and any patient in whom large fields will be anesthetized. Clinicians must be comfortable converting concentration (% w/v) to milligrams per milliliter and summing all sources of drug including topical, infiltrative, nerve block, and tumescent components over a defined interval [6,26,41].

During infiltration and nerve blocks, small-volume, incremental injection with frequent aspiration is critical to avoid inadvertent intravascular administration, especially in highly vascular regions (face, scalp) and near named vessels [19]. Buffering lidocaine with sodium bicarbonate reduces injection pain without altering systemic absorption in a clinically meaningful way [54]. Slower, less painful injections may indirectly reduce vasovagal reactions and sudden patient movement that could precipitate vascular puncture.

The inclusion of epinephrine, when not contraindicated, significantly decreases systemic uptake and prolongs block duration, enabling lower total doses for equivalent procedural coverage [16,17]. Concerns about epinephrine in end-arterial sites have been largely allayed in healthy patients, but in severely vasculopathic digits or limbs, its use should remain conservative or be avoided [2,3]. Clinicians must account for drug-drug interactions detailed in Table 4.

Monitoring intensity should be tailored to anticipated systemic exposure. Minor excisions under small-volume infiltration may require only intermittent observation. In contrast, large-field tumescent anesthesia or multiple simultaneous procedures warrant baseline and periodic blood pressure, heart rate, and pulse oximetry measurements, and some experts advocate continuous monitoring akin to moderate sedation standards [6,17]. Particular vigilance is warranted in pediatric, geriatric, and medically complex patients.

### 5.5 Recognition and Emergency Management of LAST

#### 5.5.1 Early Recognition: The Critical Window:

Timely recognition of evolving LAST is paramount; most patients exhibit a prodrome prior to seizures or cardiovascular collapse. Any sudden onset of tinnitus, circumoral numbness, metallic taste, agitation, or visual disturbance during or shortly after anesthetic administration should trigger immediate cessation of injection and focused assessment [19].

The differential diagnosis includes vasovagal syncope (pallor, bradycardia, hypotension, nausea, often triggered by needles or blood), panic or anxiety reactions (tachycardia, hyperventilation, paresthesia without objective neurologic signs), and allergic phenomena (urticaria, bronchospasm, hypotension) [55,56]. In contrast to LAST, vasovagal episodes rarely produce tinnitus, metallic taste, or focal neurologic symptoms and are promptly reversible with Trendelenburg positioning and reassurance. True IgE-mediated allergy to amide local anesthetics is exceptionally rare; most “allergic” reactions are vasovagal or pharmacologic epinephrine effects [6].

Recognition of a possible toxic prodrome should prompt stopping further anesthetic, summoning assistance, applying high-flow oxygen, establishing IV access, and preparing benzodiazepines and lipid emulsion in case of progression [19].

#### 5.5.2 The Lipid Emulsion Revolution:

The introduction of intravenous lipid emulsion therapy has transformed the prognosis of severe LAST. Initially empirically observed in animal models and then in dramatic case reports of bupivacaine-induced cardiac arrest, 20% lipid emulsion is now a central component of resuscitation algorithms [19,48].

The predominant mechanistic hypothesis is the “lipid sink” or “lipid shuttle”: the intravascular lipid phase sequesters lipophilic local anesthetic molecules away from cardiac and neuronal membranes, reducing their effective tissue concentration and facilitating redistribution to metabolically active organs such as the liver [19]. Additional proposed mechanisms include direct positive inotropy and improved mitochondrial function.

Current American Society of Regional Anesthesia and Pain Medicine (ASRA)-endorsed dosing for severe LAST in adults recommends an initial bolus of 1.5 mL/kg of 20% lipid emulsion over 1 minute, followed by a continuous infusion at 0.25 mL/kg/min, with repeat bolus and increased infusion rate (up to 0.5 mL/kg/min) if hemodynamic instability persists, to a typical upper limit of 10–12 mL/kg total [19]. Numerous case series and registry data document successful reversal of otherwise refractory cardiac arrest and rapid neurologic recovery when lipid is administered early [48].

Given its life-saving potential, 20% lipid emulsion should be immediately available in any dermatologic or aesthetic practice that performs high-dose local anesthesia, tumescent procedures, or deep regional blocks [51].

#### 5.5.3 Stepwise Management Algorithm:

The management of LAST rests on three pillars: airway and ventilation, seizure control, and cardiovascular support, integrated with lipid therapy and modified ACLS protocols [19,56].

Airway management is primary. Hypoxia and acidosis potentiate cardiotoxicity and lower seizure threshold; immediate administration of 100% oxygen and assisted ventilation with bag–valve–mask are essential, with early consideration of endotracheal intubation if consciousness is impaired [19].

Seizures should be treated promptly with benzodiazepines (e.g. midazolam 0.05–0.1 mg/kg IV, diazepam 0.1 mg/kg) or, if unavailable, small doses of propofol in hemodynamically stable patients [19]. Large boluses of propofol are discouraged in hemodynamically fragile patients due to its myocardial depressant effects.

For cardiovascular collapse, standard ACLS algorithms apply but with critical modifications. Epinephrine, if needed, should be used in reduced doses (e.g. 10–100 μg boluses rather than 1 mg) to avoid exacerbating arrhythmias or increasing myocardial oxygen demand [19]. Vasopressin, additional bolus lidocaine, and other class I anti-arrhythmics are contraindicated, as they may worsen sodium-channel blockade [48]. High-quality chest compressions should continue as lipid emulsion is administered; prolonged resuscitation efforts are justified because successful neurologic recovery after extended cardiac arrest has been documented when lipid therapy is employed [19].

In less dramatic presentations e.g. isolated seizures without hemodynamic compromise, supportive care and lipid may still be indicated if the total dose or agent (e.g. bupivacaine) portends a risk of delayed cardiac decompensation. Close monitoring for several hours is mandatory, as recurrent events can occur as tissue-bound anesthetic redistributes.

#### 5.5.4 Office Preparedness: The LAST Kit:

Preparedness in the dermatologic office environment is central to translating these principles into outcomes. A dedicated “LAST kit” should be assembled and maintained, typically including 20% lipid emulsion (at least 500 mL), appropriately sized IV cannulas and tubing, benzodiazepines, airway adjuncts (oropharyngeal airways, bag–valve–mask), supplemental oxygen delivery systems, a defibrillator, and a printed or laminated stepwise management algorithm with dosing tables [51,56]. For practices that perform end-arterial blocks, inclusion of phentolamine for digital ischemia reversal is also advisable [2,3].

Lipid emulsion should be stored according to manufacturer recommendations, readily accessible in procedural areas, and monitored for expiration; replacing a 500 mL bag at least every 24 months, or earlier if the labeled expiry date is sooner, is inexpensive relative to the potential benefit [57].

Equally important is staff training. Periodic simulation-based drills, in which teams rehearse recognizing prodromal LAST, initiating airway support, and preparing lipid, significantly reduce time-to-treatment and improve adherence to protocols, based on experience from anesthesiology and emergency medicine [56]. In dermatology, where LAST events are infrequent, such simulations are arguably the only practical way to ensure that response pathways are retained and executable under stress.

Collectively, these data and frameworks support the central thesis that in dermatologic practice, LAST has shifted from an unpredictable catastrophe to a rare but manageable complication, if dosing is rational, patient and drug factors are appreciated, and teams are trained and equipped to respond swiftly.

## 6. Special populations: tailored approaches

### 6.1 Pediatric patients

Pediatric pharmacokinetics and pharmacodynamics differ substantially from adults and amplify both the benefits and risks of local anesthetics. Infants and young children have a higher surface-area-to-weight ratio, reduced levels of α1-acid glycoprotein, and immature hepatic enzyme systems, leading to higher free fractions and slower clearance of amide anesthetics such as lidocaine and prilocaine [6]. Because barrier disruption in atopic dermatitis and other pediatric dermatoses amplifies absorption (Section IV), topical doses and contact times must be reduced relative to intact-skin protocols [9].

Regulatory labeling reflects this vulnerability. EMLA (lidocaine 2.5% / prilocaine 2.5%) carries a methemoglobinemia warning and should not be used in: (1) preterm neonates with a gestational age <37 weeks, or (2) infants younger than 12 months who are receiving other methemoglobin-inducing drugs; neonates and infants younger than 3 months are particularly susceptible to prilocaine-associated methemoglobinemia because of immature MetHb-reducing pathways. Infants <6 months, with immature MetHb-reducing capacity, are especially vulnerable to prilocaine- and benzocaine-induced methemoglobinemia (Section 5.3) [13,14]. Age-specific intact-skin EMLA limits are summarized in Table 3; on diseased skin (for example, atopic dermatitis or chronic leg ulcers), we restrict both total dose and application time to values below those used on intact skin, in line with studies showing faster absorption and higher local and systemic concentrations on eczematous and ulcerated skin [47].

For infiltrative anesthesia, weight-based dosing must be non-negotiable. Standard maximum doses for lidocaine should be treated as ceilings, not targets, in children: weight-based infiltrative dosing should not exceed 4.5 mg/kg without epinephrine (some pediatric dental guidelines cite 4.4 mg/kg) or 7 mg/kg with epinephrine, and in infants and toddlers we avoid approaching these maxima [18]. Using more dilute solutions (0.25–0.5% lidocaine with epinephrine) allows coverage of larger fields with lower total drug load and reduced injection pain, while maintaining adequate anesthesia for cutaneous surgery [49,50]. Buffered and warmed solutions further attenuate injection pain and can be combined with very small-gauge needles [52,58].

In common pediatric procedures, a staged, layered strategy is usually safest. A thin layer of topical lidocaine (without prilocaine), applied to intact skin over a limited area for a brief period shorter than the standard adult application time, can blunt the initial needle sting [13,14]. On acutely inflamed or eczematous skin, we either avoid topical anesthetics altogether or use substantially lower doses and shorter application times than standard adult intact-skin protocols, favoring dilute infiltrative anesthesia for larger or highly inflamed fields, given the accelerated and enhanced absorption documented on diseased skin.

Parental anxiety is frequently as consequential as the child’s pain. Transparent counselling and informed consent in pediatrics should explicitly address off-label topical use on inflamed skin, the rare but real possibility of methemoglobinemia or seizures with prilocaine- and benzocaine-containing products, and the signs that would prompt emergent evaluation after discharge [52].

### 6.2 Geriatric and frail patients

In older adults, the pharmacology of local anesthetics is shaped less by chronological age than by cumulative organ dysfunction, comorbidity, and frailty. Amide anesthetics, including lidocaine and bupivacaine, rely on hepatic cytochrome P450 metabolism; aging is associated with reduced hepatic blood flow, diminished metabolic capacity, and lower plasma albumin and α1-acid glycoprotein concentrations, increasing the unbound fraction of drug and prolonging elimination [6]. Concomitant medications such as non-selective β-blockers, calcium channel blockers, and certain SSRIs further slow clearance or reduce cardiac output, thereby increasing area-under-the-curve exposure for a given infiltrated dose [6–8].

Frailty indices, which integrate functional status, comorbidity burden, and nutritional reserve, likely predict anesthetic risk more accurately than age alone. A frail octogenarian with congestive heart failure and cirrhosis will have markedly reduced lidocaine clearance and little physiologic reserve to tolerate even transient CNS or cardiovascular depression, whereas a robust septuagenarian may safely receive near-standard doses. Yet current dermatologic guidelines do not incorporate formal frailty assessments, and dosing remains largely anchored to adult mg/kg limits [6]. High-impact practice should move toward individualized dosing that treats advanced frailty as a relative “dose-reducing comorbidity,” analogous to hepatic insufficiency.

Pragmatically, in elderly patients with significant frailty, hepatic dysfunction, or polypharmacy that impairs clearance, we limit lidocaine to a maximum of 4–5 mg/kg, even though standard adult limits for lidocaine with epinephrine allow doses up to 7 mg/kg, and we reduce other amide doses analogously [6]. Preferential use of more dilute solutions (0.25–0.5% lidocaine with epinephrine) and smaller total volumes can often achieve adequate field anesthesia in atrophic geriatric dermis, which allows wider spread of injectate [49]. Epinephrine remains useful for reducing systemic uptake and improving hemostasis, but transient tachycardia and blood pressure elevations may unmask coronary insufficiency; cautious titration and avoidance of large, rapid boluses are warranted in patients with unstable coronary disease or significant arrhythmias [6].

Monitoring thresholds should be lower in frail elders. For any procedure requiring moderate-to-large volumes of anesthetic, or incorporating tumescent technique, baseline and interval vital signs and pulse oximetry are appropriate; continuous ECG monitoring is reasonable when doses approach the upper end of the reduced geriatric range, or in those with structural heart disease [51]. Cognitive changes, dizziness, or new confusion in the hours after a procedure should trigger evaluation for subclinical systemic toxicity, which appears at lower plasma thresholds in frail patients than in healthy adults [6].

## 7. Procedural techniques influencing safety

### 7.1 Tumescent technique: the high-volume safety paradigm

Tumescent anesthesia exemplifies how procedural technique can recast toxicity risk. By combining extreme dilution of lidocaine (0.05–0.1%) with epinephrine 1:1,000,000 in large volumes instilled into subcutaneous fat until tissues are firm, tumescent infiltration produces profound regional anesthesia, hydrodissection, and hemostasis while dramatically slowing systemic uptake [17]. Pharmacokinetic studies show that within evidence-based mg/kg ranges, tumescent anesthesia yields peak serum lidocaine concentrations well below the 6 μg/mL threshold for mild CNS toxicity, with peaks delayed for many hours after infiltration [26]. Epidemiologic reviews encompassing 396,457 tumescent liposuction procedures performed with tumescent anesthesia as the sole anesthetic technique and following contemporary dosing protocols have not identified a single tumescent-anesthesia–associated death [59,60].

Safety in this paradigm depends critically on infusion rate and infiltration pattern. Slow, staged instillation (often via a pump or pressure bag) into a fan or grid of subcutaneous tunnels allows epinephrine’s vasoconstriction to develop as lidocaine is deposited, limiting early systemic escape and flattening the plasma concentration–time curve [17,26,41]. Segmenting very large fields (for example, tumescing one limb or oncologic field, completing surgery, then tumescing the next) further reduces peak levels by distributing absorption over time. The same principles underlie the successful extension of tumescent anesthesia beyond liposuction to extensive Mohs surgery, large excisions and flap/graft reconstructions, full-face laser resurfacing, hair transplantation, axillary hyperhidrosis surgery, and even burn debridement [39,51].

Despite its “mega-dose” appearance on paper, tumescent anesthesia should be viewed as a safety-enhancing technique in high-risk dermatologic settings where large areas must be anesthetized and topical strategies would entail unpredictable, rapid absorption through compromised skin [9,17]. Its principal procedural caveat is architectural distortion: all margins and anatomic landmarks must be carefully marked before tumescence [17]. Key practice recommendations across these domains are summarized in table 5.

## 8. Conclusion

In summary, when local anesthetic choice, dose, and technique are matched to vascular reserve, barrier integrity, and host pharmacokinetics, even traditionally high-risk dermatologic scenarios can be managed with very low rates of serious harm. The main threats arise not from routine use but from predictable amplifiers such as severe vasculopathy, extensive barrier loss, compressed cumulative dosing, and unrecognized drug interactions. This shift from dogma to risk-stratified utilization heightens rather than relaxes the obligation for preparedness: rigorous dose calculation and documentation, systematic medication review, context-sensitive selection of agent and route, and office-level readiness with phentolamine and 20 percent lipid emulsion rescue supported by checklists and team training.

## Figures and Tables

**Figure 1:**
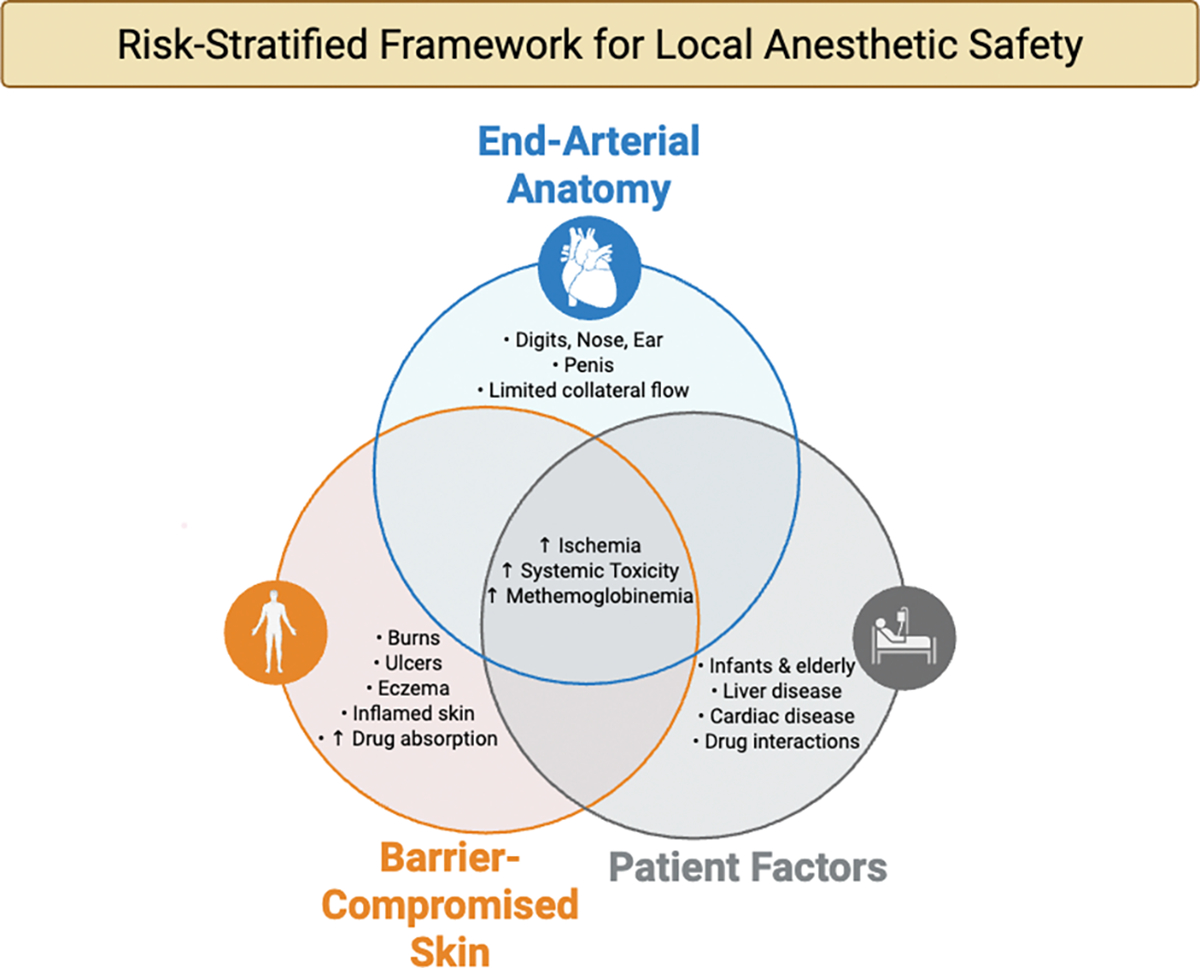
Conceptual framework illustrating the three interacting domains, anatomic site (end-arterial circulation), skin barrier integrity, and patient-specific factors, that collectively determine the risk of local anesthetic toxicity in dermatologic surgery.

**Figure 2:**
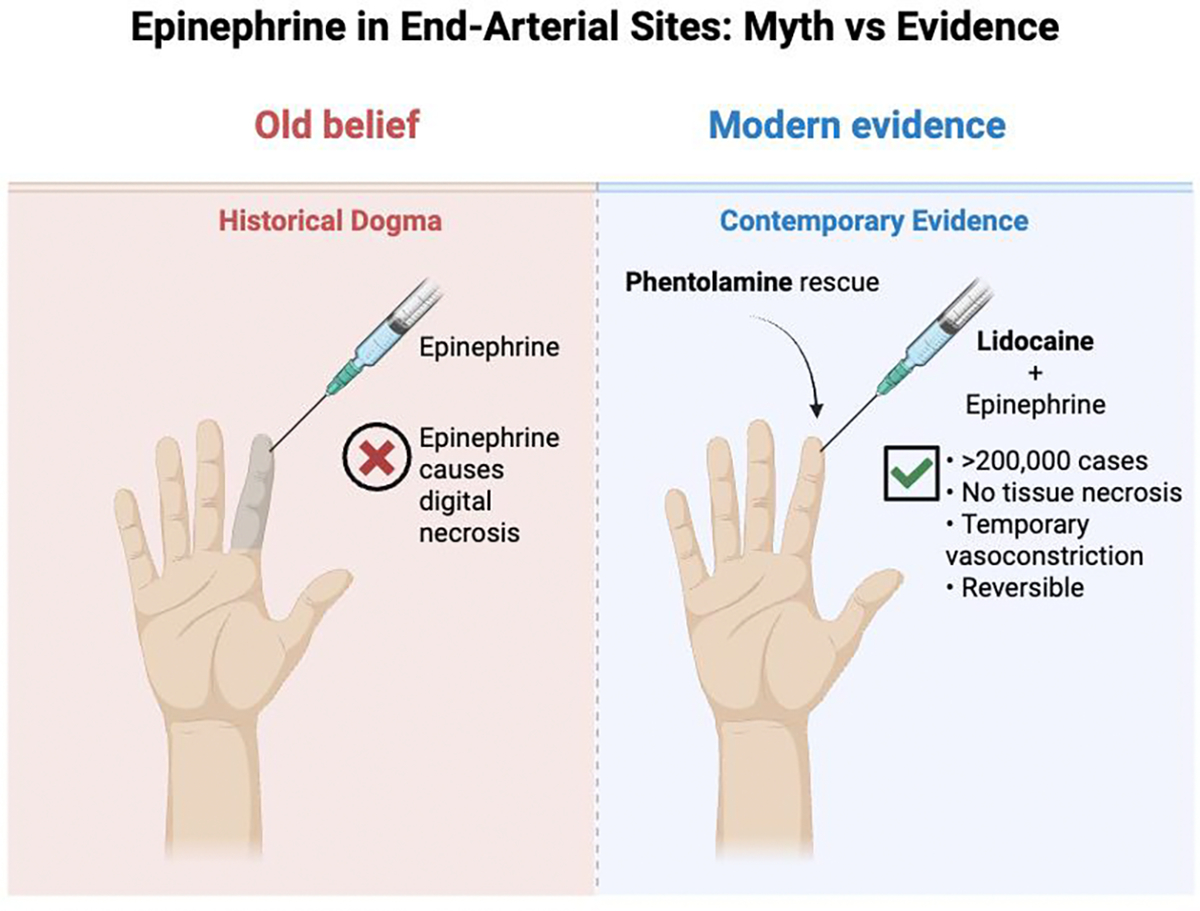
Visual comparison of historical contraindications versus contemporary evidence regarding epinephrine use in end-arterial sites, demonstrating transient, reversible vasoconstriction without tissue necrosis when used appropriately.

**Figure 3:**
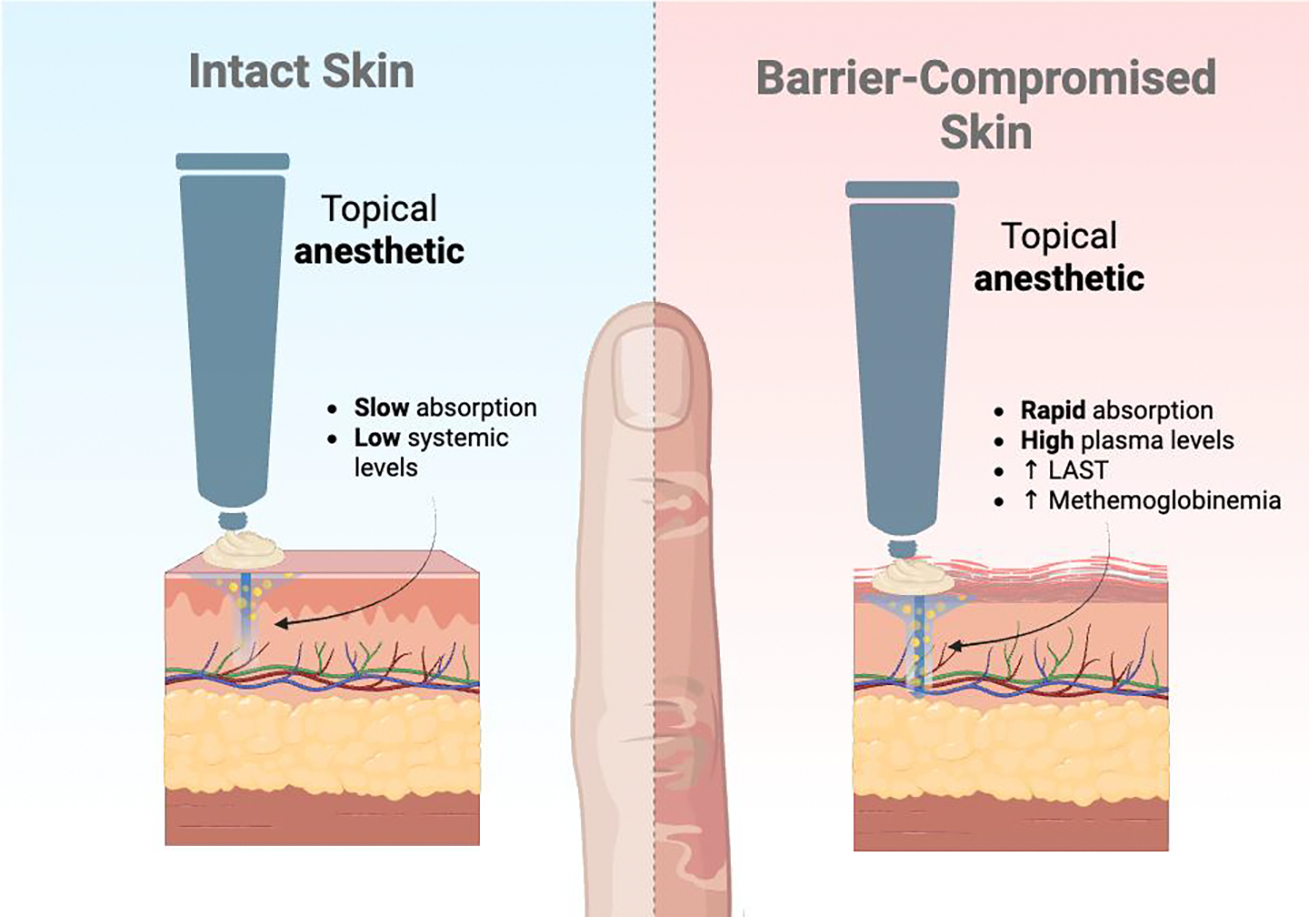
Comparison of topical local anesthetic absorption through intact versus barrier-compromised skin, illustrating accelerated systemic uptake and increased risk of toxicity when the epidermal barrier is disrupted.

**Figure 4:**
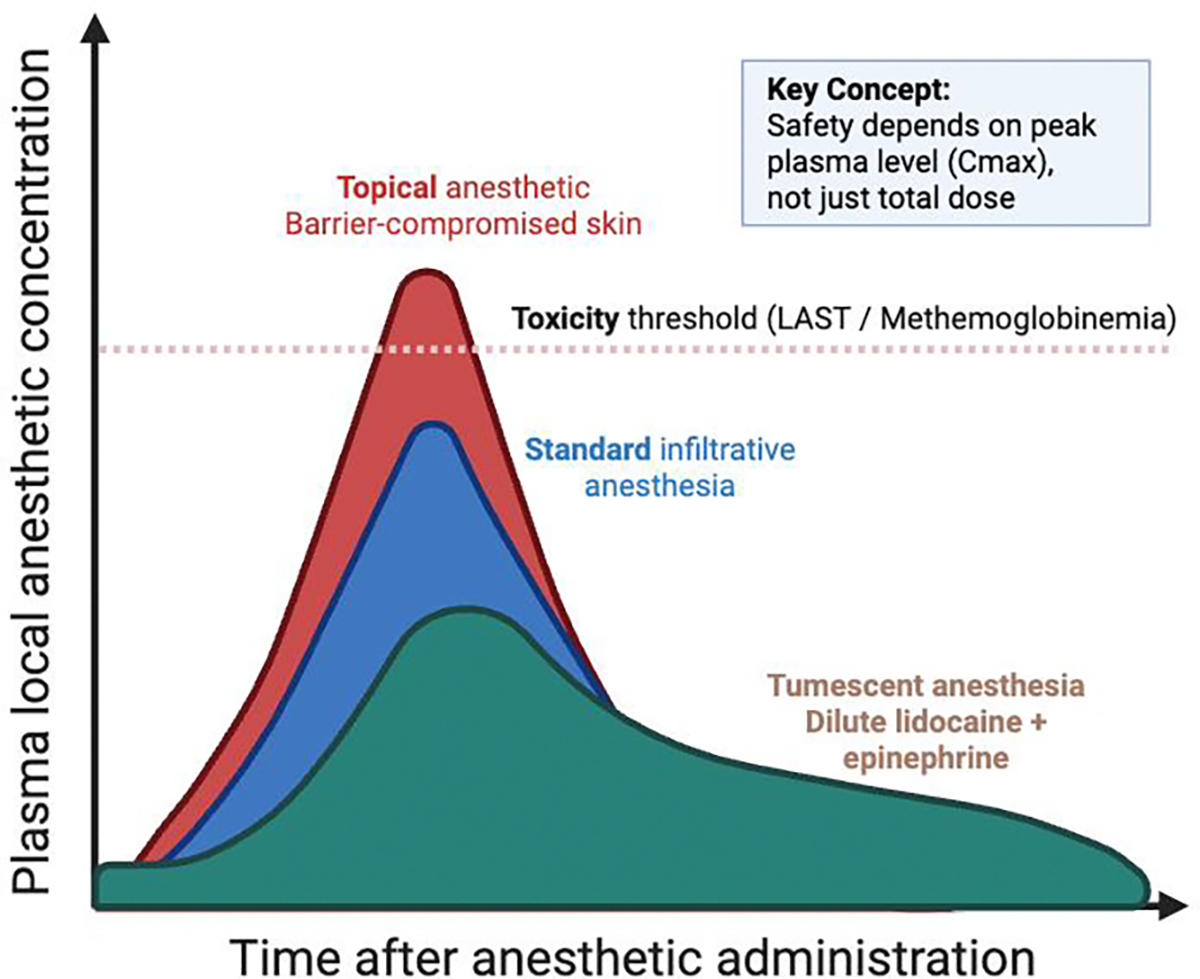
Integrated pharmacokinetic and mechanistic model demonstrating how delivery method, skin barrier integrity, vascular absorption, and epinephrine-mediated vasoconstriction influence plasma local anesthetic concentrations and systemic toxicity risk.

**Table 1:** Pharmacologic properties, clinical uses, and maximum doses of local anesthetics used in dermatology.

Agent (class)	pKa	Relative lipid solubility / potency	Protein binding (%)	Typical dermatologic use / concentration	Approximate duration (plain / with epinephrine)	Maximum adult dose† plain (mg/kg; absolute)	Maximum adult dose† with epinephrine (mg/kg; absolute)	Dominant toxicity / key notes
**Lidocaine (amide)**	7.9	Intermediate	65	Infiltration 0.5–2%; tumescent 0.05–0.1%; topical 4–5%	60–120 min / 120–360 min	4.5–5 mg/kg (≤300 mg) [18].	7 mg/kg (≤500 mg) [18].	Workhorse agent; CNS excitation with seizures usually precedes cardiovascular collapse; systemic levels increased with barrier compromise and CYP inhibition; rare true allergy [9, 19].
**Bupivacaine (amide)**	8.1	High	95	Peripheral nerve blocks 0.25–0.5%	240–480 min / similar	2.0–2.5 mg/kg (maximum 175 mg for a single dose in office-based dermatology)[20, 21].	3.0 mg/kg (maximum 225 mg; do not exceed 400 mg in 24 hours) )[20, 21].	Long-acting; marked cardiotoxicity with risk of ventricular arrhythmias and arrest at modest plasma levels; long half-life increases risk with repeat or large-volume dosing (use small volumes, avoid cumulative dosing) [9].
**Ropivacaine (amide)**	8.1	High (slightly less than bupivacaine)	94–95	Long-acting nerve blocks 0.2–0.5% (rarely used in cutaneous-only dermatology)	180–360 min / modestly prolonged with epinephrine	3.0 mg/kg (adult maximum; use lower doses in frail or comorbid patients) [20, 21].	3.0 mg/kg (adult maximum; use lower doses in frail or comorbid patients) [20, 21].	Lower cardiotoxicity than bupivacaine but same qualitative pattern of CNS/CV depression; avoid high cumulative doses and use in settings with resuscitative capacity.
**Mepivacaine (amide)**	7.6	Intermediate	75	Infiltration or blocks 1–2%; often without epinephrine	90–180 min / 120–360 min [16, 22].	5–6 mg/kg (≤400 mg) [6].	5–6 mg/kg (≤400 mg); limited benefit from epinephrine (many sources do not increase the maximum) [6].	CNS toxicity profile similar to lidocaine; relatively minimal vasodilation, making it useful when vasoconstrictors are undesirable.
**Prilocaine (amide)**	7.9	Intermediate	55–65	EMLA (2.5%); occasional infiltration 0.5–1%	60–120 min / prolonged with epinephrine	6.0 mg/kg (maximum 400 mg in healthy adults)[23].	8.0 mg/kg (maximum 500 mg in healthy adults)[23].	Methemoglobinemia via o-toluidine metabolite, especially in infants, G6PD deficiency, and large-area or mucosal/ulcer applications; CNS/CV toxicity less prominent at doses causing clinically significant MetHb [13, 14].
**Procaine (ester)**	8.9	Low	6	Historically used for infiltration; now largely obsolete in dermatology	45–60 min / 60–90 min [16, 22].	7 mg/kg (≤350 mg) [16, 22].	10 mg/kg (≤600 mg) [16, 22].	Rapid hydrolysis by pseudocholinesterase; relatively low systemic toxicity; PABA-mediated allergy more frequent; historical digital necrosis described in non-standard mixtures and conditions [2, 3].
**Tetracaine (ester)**	8.5	Very high	75–76	Topical 0.5–4% (alone or in TAC/LET); ocular drops	120–180 min topically	1.0 mg/kg injectable (systemic use is rare in dermatology; 1.5 mg/kg reported when combined with epinephrine in anesthesia literature)[16, 22].	1.5 mg/kg (maximum adult dose with epinephrine in systemic use; injectable tetracaine with epinephrine is rarely required in dermatology) [16, 22, 24]	Very potent; significant CNS and CV toxicity when systemically absorbed; serious events documented with high-concentration compounded creams; increased allergy risk due to PABA metabolite [25].
**Benzocaine (ester)**	3.5 (poorly water-soluble)	High topical potency	63–76	Topical 5–20% gels/sprays (mucosa, ulcers)	Very short topical duration	No reliable mg/kg limit‡; use minimal amount, especially on mucosa [13, 14].	No reliable mg/kg limit‡; not used with epinephrine [13, 14].	Strong association with methemoglobinemia, particularly in infants and with mucosal application; multiple regulatory safety warnings; should be avoided in infants and G6PD deficiency and used sparingly in adults [13, 14].
**Cocaine (ester)**	8.7	High	92	Topical 4–10% for nasal mucosa (ENT); rarely dermatologic	30–60 min	1.5–3 mg/kg (≤200 mg adult) [25].	Not routinely combined with epinephrine (intrinsic vasoconstrictor).	Intrinsic vasoconstrictor and sympathomimetic; systemic toxicity includes hypertension, coronary vasospasm, and arrhythmias; largely replaced in dermatology by lidocaine–epinephrine mixtures [25].

† Maximum doses are approximate values for **healthy adults** receiving standard **infiltrative** anesthesia with normal hepatic and cardiac function; pediatric, frail, and comorbid patients require lower thresholds [6,18]. Tumescent anesthesia follows distinct, higher dose limits based on dilute concentrations and pharmacokinetics [26].

‡ For benzocaine, systemic exposure is highly variable and serious methemoglobinemia has occurred after apparently modest doses; no safe mg/kg ceiling can be defined. Use the smallest effective amount on the smallest possible mucosal area and avoid in infants and high-risk hosts [13,14].

**Table 2:** Summary of evidence for epinephrine safety in end-arterial sites.

Study / source	Design	Anatomic site(s)	Anesthetic formulation	Sample size	Comorbidities included	Ischemic complications attributed to epinephrine	Key findings
Ilicki 2015 systematic review [27].	Systematic review of 23 clinical studies	Fingers, toes	Lidocaine ± bupivacaine with epinephrine 1:100,000–1:200,000	2,797 digital blocks	Mixed; many included diabetics, hypertensives	0 cases of digital necrosis or amputation	No evidence of epinephrine-induced gangrene with modern concentrations; supports abandonment of blanket contraindication.
Denkler WALANT and Lalonde Dalhousie multicenter series [2, 28].	Retrospective and prospective cohorts	Fingers, hand	Lidocaine 1–2% with epinephrine 1:100,000	3,110 consecutive elective finger and hand procedures; additional WALANT cohorts in this review together contribute several thousand further cases, all without epinephrine-attributed digital necrosis [2, 28]	Excluded severe ischemia; included diabetics, smokers	0 digital necroses; no phentolamine required in large majority	Demonstrated safety and operative advantages (no tourniquet, excellent hemostasis) in routine use.
Prospective digital surgery series [2].	Prospective cohort	Fingers	Lidocaine 1% with epinephrine 1:100,000	1,340 surgeries	Common comorbidities allowed; severe ischemia excluded	0 ischemic events, no necrosis	Validated clinical safety in a large unselected cohort.
Auto-injector injury case series [2, 3].	Case series, literature review	Fingers	Epinephrine 1:1,000 (0.3–0.5 mg) accidental injection	Literature review: 59 cases; Poison-center cohort: 213 digital injections (127 with documented follow-up).	Many healthy; some smokers	0 necroses in both series; 4 transient ischemic episodes in the poison-center cohort, all resolving completely, 2 within 2 hours.	Even massive local epinephrine rarely causes tissue loss, underscoring safety margin at dilute concentrations.
Ear and nose tumescent series [29].	Prospective observational	Ear, nose	Tumescent lidocaine 0.1% with epinephrine 1:200,000	>10,000 dermatologic/ENT cases	General population; high-risk vasculopathy uncommon	0 flap or skin necroses attributable to epinephrine	Marked flow reduction, with a 69% decrease in dermal blood flow and a 42% decrease in arterial inflow documented by laser Doppler and acral photoplethysmography, but full recovery without tissue damage[29].
Penile block cohort [33].	Prospective cohort	Penis	Lidocaine with epinephrine (typically 1:100,000)	95 patients undergoing penile surgery under local anesthetic with epinephrine additive; no ischemic or erectile complications reported [2].	Not specified; major vasculopathies likely excluded	0 ischemic or erectile complications	Supports safety of epinephrine in richly vascularized penile tissue.
Case reports in Raynaud’s / scleroderma [1, 2].	Single-patient reports	Fingers	Lidocaine with epinephrine 1:100,000	Individual cases	Known vasospastic or connective tissue disease	Prolonged ischemia, blistering; rare necrosis with confounders	Suggest heightened susceptibility in severe vasospastic/vasculitic disease; basis for relative contraindication.

**Table 3:** Topical local anesthetics on intact and barrier-compromised skin: pharmacokinetics, recommended maximum exposures, and preferred alternatives for extensive fields.

Clinical scenario / patient & barrier status	Agent / topical regimen	Pharmacokinetic data (peak levels and time to peak, where available)	Recommended maximum exposure per session (adult unless stated)	Key observations / caveats
**Adult, intact skin (thigh)**	EMLA 2.5%/2.5%, up to 60 g on 400 cm^2^ of intact thigh skin under occlusion for 3 hours (adult volunteers)	After application of 60 g of EMLA to 400 cm^2^ of intact adult thigh skin for 3 h, mean peak plasma concentrations are 0.12 μg/mL for lidocaine and 0.07 μg/mL for prilocaine, with peaks occurring within 4 h of application; these levels are more than 40-fold lower than the 5 μg/mL plasma concentration commonly associated with systemic toxicity [43,44].	Up to 60 g on ≤400 cm^2^ of intact skin under occlusion for up to 3 h in healthy adults, in line with product labeling [44].	Predictable sub-toxic exposure despite large dose and area; avoid exceeding 60 g or 400 cm^2^ in a single session; consider cumulative dose if repeated within the same day [6,43].
**Adult, chronic venous or pressure ulcer**	EMLA 2.5%/2.5%; PK study regimen: 5–10 g on 50–100 cm^2^ ulcer, occluded for 24 h, with repeated-dose regimens over 10–15 applications	After a single 24-h application of 5–10 g of EMLA to leg ulcers measuring 50–100 cm^2^, maximum plasma lidocaine concentrations range from 0.18 to 0.70 μg/mL and prilocaine from 0.06 to 0.28 μg/mL, with peak levels occurring 2–4 h after application; repeated applications of 2–10 g for 30–60 min on ulcers up to 62 cm^2^, up to 15 sessions over one month, do not produce measurable accumulation of either anesthetic in plasma [5,44].	5–10 g on ulcers up to 100 cm^2^ for 30–60 min before debridement, once daily for up to 10 days, staying within the studied regimens for 24-h and repeated applications [5,38,44].	Safe sub-toxic levels even with prolonged and repeated exposure on ulcer beds; prefer 5 g rather than 10 g in frail elderly or those with hepatic impairment; avoid multiple large-area applications within 24 h [5,6].
**Adult, localized partial-thickness burns (≤25–50 cm^2^)**	EMLA 2.5%/2.5% ≤5 g under short occlusion; or lidocaine 4–5% cream on small burn areas	After applying 5 g of EMLA (containing 125 mg lidocaine and 125 mg prilocaine) to 25 cm^2^ of second-degree burns for 30 min, maximum observed peak plasma concentrations are 0.412 μg/mL for lidocaine and 0.206 μg/mL for prilocaine, with peaks reached 15–30 min after application; combined concentrations remain at least ten-fold below the 5–10 μg/mL range associated with systemic toxicity, and no serious systemic adverse events were reported [45].	≤5 g on ≤25–50 cm^2^ for 30–45 min, with removal before debridement or dressing changes [6,45].	Safe for localized burn analgesia when area and dose are restricted; do not extrapolate to large burns; for more extensive burns, staged fields or infiltrative/tumescent techniques are preferred [4,9,45].
**Adult, extensive partial-thickness burns (28% total body surface area, high-risk scenario)**	5% lidocaine cream applied at 1 mg/cm^2^ (4.5 g total) to a 28% total body surface area partial-thickness burn [4].	In the reported case, plasma lidocaine concentration was 5.8 μg/mL at each measurement from 15 to 240 min after application, within the range associated with systemic CNS toxicity [4].	High-dose 5% lidocaine over large burn surface areas is **not recommended**; no safe maximum can be defined for such extensive fields.	Illustrative of life-threatening systemic exposure when high-strength lidocaine is used over large burns; supports guidance to avoid treating large contiguous burn areas topically and to favor staged fields or dilute infiltrative/tumescent anesthesia for extensive burns [4,6].
**Adult, atopic dermatitis lesions (localized)**	EMLA 2.5%/2.5% thin layer on small eczematous areas	Quantitative Cmax values were not reported, but in atopic dermatitis EMLA produces effective anesthesia on eczematous skin within 5–15 min, compared with 30–60 min on normal skin; the accentuated blanch–erythema response suggests increased absorption for a given dose [9,37,43].	Not formally defined in trials; in practice, a thin layer applied to the minimal necessary area with a contact time of 15–30 min, keeping well below intact-skin dose and area limits, is generally adequate [37,44].	Barrier disruption and inflamed microvasculature markedly increase absorption; avoid occlusion; use the smallest effective area and duration; avoid prilocaine-containing preparations in infants or patients at high methemoglobin risk [14,34,37].
**Adult, extensive erosive dermatoses (non-burn)**	Lidocaine-only 4–5% cream, applied in multiple small fields	Systematic PK data are lacking; by extrapolation from diseased-skin studies and case series, absorption is expected to be significantly enhanced compared with intact skin [6,9,43].	As a conservative upper limit, restrict total lidocaine-only cream to no more than 10 g per session, divided into multiple small fields each receiving no more than 5 g and removed after 20–30 min [6,9].	Avoid prilocaine and benzocaine because of methemoglobin risk; avoid occlusion; for very large surface areas, stage treatment over multiple days rather than a single large-area application [6,9].
**Ablated / laser-resurfaced skin (adult case report)**	30% lidocaine gel applied over fractional laser-resurfaced field [35].	Exact Cmax was not reported; life-threatening CNS toxicity with seizures occurred within less than 2 h of application [35].	No safe mg/kg or surface-area limit is established for 30% lidocaine on ablated skin; such high-concentration compounded formulations over large, denuded fields should be considered unsafe.	Extreme example of rapid, high systemic lidocaine uptake from ablated skin; underpins recommendations to avoid high-strength compounded topicals on denuded or laser-resurfaced skin and to use dilute infiltrative/tumescent anesthesia for large resurfacing fields [6, 35].
**Infants 0–3 months, intact skin**	EMLA 2.5%/2.5% (if used at all)	Detailed PK data are limited in this age group; infants have reduced cytochrome b5–dependent methemoglobin-reducing capacity and higher relative systemic exposure for a given area, which increases susceptibility to prilocaine-induced methemoglobinemia [44,46,47].	≤1 g on ≤10 cm^2^ for ≤1 h on intact skin; many centers avoid EMLA entirely in this age group [44,47].	High risk of methemoglobinemia; avoid use on diseased or broken skin; consider alternatives such as brief, carefully dosed lidocaine infiltration or non-pharmacologic analgesia [14,36,47].
**Infants 3–12 months, intact skin**	EMLA 2.5%/2.5%	PK studies in older infants and children show low lidocaine and prilocaine plasma concentrations at recommended doses, but higher surface-area-to-body-weight ratio and immature metabolism still increase relative exposure [44,46,47].	≤2 g on ≤20 cm^2^ for ≤1 h on intact skin, in line with pediatric labeling [44].	Do not use on eczematous or ulcerated skin; avoid repeated large-area applications; consider lidocaine-only creams for any barrier-compromised sites [14,34,36].
**Children >12 months, localized barrier-compromised skin (eczema, small ulcers)**	Lidocaine-only 4–5% cream	Quantitative PK data are limited in this specific scenario; absorption is increased relative to intact skin, and clinical effect is typically achieved within 20–30 min, consistent with diseased-skin absorption and pediatric EMLA data [37,43,46].	Pragmatically ≤0.5 g per 10 cm^2^ for ≤20–30 min; total dose scaled by weight and kept well below adult maxima and labeled intact-skin pediatric limits [6,37,44].	Avoid prilocaine and benzocaine because of methemoglobin risk; strict avoidance of occlusion; cumulative topical and infiltrative doses must be integrated into a single weight-based calculation [6,48].
**Elderly with chronic leg ulcers**	EMLA 2.5%/2.5% applied to ulcer bed	PK is similar in pattern to younger adults with leg ulcers: after 24 h application of 5–10 g to ulcers measuring 50–100 cm^2^, lidocaine maximum plasma concentrations are 0.18–0.70 μg/mL and prilocaine 0.06–0.28 μg/mL, with peaks at 2–4 h; in frail elders, overall clearance may be slower because of reduced hepatic blood flow and polypharmacy [5,8,44].	5 g on ≤50–60 cm^2^ for 30–45 min before debridement, with a lower total dose and smaller treated area than in younger adults [5,6,8].	Reduce dose relative to younger adults to reflect slower clearance and comorbidity; consider lidocaine-only preparations in anemic or cardiopulmonary-limited patients; avoid multiple large-area applications within 24 h [5,6,8].

**Table 4:** Systemic risk modifiers for local anesthetic toxicity: concomitant medications and clinical populations.

Risk modifier or population	Mechanism of Interaction	Impact on Systemic Toxicity & Pharmacokinetics	Clinical implications and recommended practice
**Concomitant medication – Non-selective β-blockers (e.g. propranolol)**	Lowers cardiac output and hepatic blood flow; β-blockade blunts compensatory adrenergic responses [7,8].	Increases Lidocaine AUC; masked tachycardia; exaggerated bradycardia and hypotension if toxicity occurs.	Reduce maximum doses; inject incrementally; monitor hemodynamics closely, particularly with large fields or tumescent anesthesia.
**Concomitant medication – SSRIs (e.g. sertraline, fluoxetine)**	CYP3A4/2D6 inhibition; ↓ seizure threshold [9,26,41].	Slower lidocaine clearance; earlier onset of CNS symptoms at lower plasma levels.	Use conservative dosing in high-dose contexts (e.g. tumescent); warn patients about possible delayed mild CNS symptoms; consider lower cumulative mg/kg ceilings.
**Concomitant medication – TCAs (e.g. amitriptyline)**	Sodium-channel blockade; CYP inhibition; catecholamine reuptake inhibition [9].	Additive cardiac conduction slowing; potentiation of epinephrine effects; lower seizure threshold.	Avoid high cumulative LA doses; monitor ECG when large blocks are performed; use epinephrine cautiously and avoid very high concentrations.
**Concomitant medication – Class I anti-arrhythmics (e.g. mexiletine)**	Structural/functional analogues of lidocaine, with additional sodium-channel blockade [9].	Additive risk of conduction block and arrhythmias when combined with lidocaine or other amide anesthetics.	Minimize lidocaine dose; prefer shorter-acting agents; consider cardiology input for high-risk cases; avoid stacking multiple sodium-channel blockers.
**Concomitant medication – Amiodarone**	CYP inhibition; negative inotropy; prolonged repolarization.	Potential for prolonged lidocaine half-life and exaggerated myocardial depression or conduction disturbances.	Use lower dosing thresholds; continuous monitoring (BP, ECG, SpO₂) for large blocks or tumescent anesthesia; be cautious with bupivacaine and other cardiotoxic agents.
**Concomitant medication – Benzodiazepines**	Mild CYP3A4 inhibition; GABAergic CNS depression [26,41].	Slightly reduced lidocaine clearance but elevated seizure threshold; may attenuate CNS manifestations of toxicity.	Generally favorable for seizure prophylaxis in high-risk LAST scenarios; no major dose adjustment needed solely for this interaction, but do not allow benzodiazepines to mask evolving cardiovascular toxicity.
**Population / comorbidity – Neonates and infants <3 months**	High surface-area-to-weight ratio; immature hepatic metabolism and methemoglobin reductase; low α1-acid glycoprotein [6,13,14].	Higher free fraction and slower clearance of amide anesthetics; marked susceptibility to prilocaine- and benzocaine-induced methemoglobinemia.	Avoid prilocaine- and benzocaine-containing topicals; if infiltrative lidocaine is required, do not exceed 3 mg/kg of plain lidocaine (reduced from the adult maximum of 4.5 mg/kg due to immature metabolism) and use dilute solutions; favor non-pharmacologic analgesia and very brief, carefully dosed lidocaine creams on intact skin; no home use of high-strength compounded topicals [13,14,25].
**Population / comorbidity – Children ≥3 months**	Immature but rapidly maturing hepatic clearance; frequent barrier disorders (eczema, molluscum) [9].	Increased systemic uptake from diseased skin; narrower margin between therapeutic and toxic levels than adults.	Use 0.25–0.5% lidocaine with epinephrine; limit the total infiltrative lidocaine dose to no more than 4.5 mg/kg without epinephrine or 7 mg/kg with epinephrine, and stay below these maxima when treating large fields or inflamed skin; combine limited-area topical lidocaine (no prilocaine) with buffered, warmed minimal-volume injections; avoid occlusion on diseased skin; provide explicit parental counselling on signs of toxicity [13,14,37,49].
**Population / comorbidity – Frail geriatric patients**	Reduced hepatic blood flow and protein binding; age-related decrease in metabolic capacity; polypharmacy (β-blockers, SSRIs, others) slows clearance and masks adrenergic warning signs [6–8].	Higher unbound drug fraction; slower elimination; lower physiologic reserve for hypotension or arrhythmias.	Reduce the total amide dose by 20% compared with standard adult maximum doses; prefer dilute solutions; avoid large, rapid boluses; use buffered dilute lidocaine–epinephrine with slow injection; consider vital-sign and pulse-oximetry monitoring for larger cases; treat frailty as equivalent to hepatic/cardiac impairment when planning dosing [6,51].
**Population / comorbidity – Diabetes and mild–moderate PAD with intact pulses**	Microangiopathy but preserved macrovascular flow; good tolerance of transient pharmacologic vasoconstriction in the absence of critical ischemia [2,3].	Generally similar systemic LA handling to non-diabetics; digital perfusion usually adequate for dilute epinephrine.	Standard mg/kg dosing acceptable; no routine need to reduce epinephrine concentration beyond 1:100,000–1:200,000; dilute epinephrine-containing digital and field blocks are appropriate; avoid tourniquet when WALANT is feasible; monitor perfusion clinically; have phentolamine available for unexpected prolonged blanching [2,3].
**Population / comorbidity – Critical ischemia, Buerger’s disease, severe Raynaud’s**	Severely limited collateral flow and/or exaggerated vasospasm; structurally or functionally compromised digital/microvascular circulation [1,2].	High risk that α-adrenergic vasoconstriction (epinephrine) will tip precarious perfusion into critical ischemia; increased risk of ischemic complications at end-arterial sites.	Avoid epinephrine at end-arterial sites; use plain lidocaine at reduced mg/kg and volume; prefer plain infiltrative or more proximal nerve blocks with careful aspiration and gentle injection; maintain warmth; have phentolamine available if inadvertent or unavoidable epinephrine exposure occurs [2,3].
**Population / comorbidity – Immunocompromised / transplant patients**	Often multi-morbid with variable hepatic and renal function; increased infection risk from chronic immunosuppression [6].	LA handling primarily determined by hepatic and cardiac status rather than immune status per se; infection risk elevated for any injection or topical over ulcers/dermatitis.	Adjust doses according to hepatic and cardiac function rather than immunosuppression alone; no specific LA class-based dose change required; use standard lidocaine–epinephrine regimens with meticulous asepsis; limit high-dose topicals on ulcers or heavily inflamed dermatitis; favor tumescent techniques for large fields to keep systemic levels low [9,17].

**Table 5:** High-risk dermatologic anesthetic contexts: principal hazards, preferred anesthetic strategies, and critical safeguards.

High-risk context	Principal hazards	Preferred anesthetic strategies	Critical safeguards
End-arterial sites (digits, nose, ear, penis)	Ischemia/necrosis; inadvertent intra-arterial injection.	Lidocaine 1–2% with epinephrine 1:100,000–1:200,000 (5–10 μg/mL) in patients with intact pulses; plain lidocaine in critical ischemia, Buerger disease, or severe Raynaud phenomenon [1–3].	Strict aspiration and slow incremental injection; careful vascular examination and documentation; phentolamine available with a protocol for epinephrine-induced ischemia (for example, 1 mg phentolamine diluted in 1 mL normal saline infiltrated at the prior epinephrine injection sites if the digit remains pale or poorly perfused beyond the expected interval) [1–3].
Barrier-compromised skin (burns, ulcers, dermatitis)	Rapid, enhanced absorption; methemoglobinemia; CNS/CV toxicity from topicals [9,25].	Topical lidocaine without prilocaine or benzocaine applied only to limited areas, not exceeding approximately 1 mg lidocaine/cm^2^ of treated burn surface as used in clinical studies, and avoiding treatment of more than about one quarter of total body surface area in a single session; avoid or sharply limit occlusion; for larger fields, prefer dilute infiltrative or tumescent anesthesia over high-dose topical therapy [37,39].	Mandatory dose and time reduction vs intact-skin protocols; no unsupervised high-concentration compounded creams; vigilance for early neurologic or cyanotic signs; prompt removal of topical if concern arises.
Large-volume or high-dose procedures (liposuction, extensive excisions, burn debridement)	Cumulative systemic dose; delayed LAST from prolonged absorption.	Tumescent anesthesia with 0.05–0.1% lidocaine plus epinephrine 1:1,000,000, delivered by slow staged infiltration; total lidocaine dose not exceeding 28 mg/kg when tumescent anesthesia is used without liposuction and 45 mg/kg when combined with liposuction, with dermatologic surgery guidelines allowing up to 55 mg/kg for liposuction under strict monitoring [17,26].	Pre-procedure dose calculation and independent double-check; continuous or interval monitoring; cumulative accounting of all lidocaine sources over 24 hours; a LAST kit with 20% lipid emulsion immediately available, using guideline-based dosing (for example, an initial 1.5 mL/kg bolus followed by infusion up to a maximum cumulative dose of 10–12 mL/kg in the first 30 minutes) [51].
Systemic toxicity risk (any patient at upper mg/kg limits or with major comorbidities)	LAST: seizures, arrhythmias, cardiovascular collapse [19].	Conservative dosing tailored to hepatic, renal, and cardiac function; epinephrine-containing solutions to slow absorption when not contraindicated; volume-sparing nerve and field blocks, ultrasound-guided in selected cases [7,8,61].	Office LAST kit with 20% lipid emulsion (including clear dosing instructions such as a 1.5 mL/kg IV bolus followed by infusion, with a maximum cumulative dose of 10–12 mL/kg), staff trained via checklists and simulation; prompt recognition of prodromal CNS signs; immediate cessation of injection and initiation of lipid rescue for serious events [19,62].
Special populations (pediatric, frail geriatric, immunocompromised)	Age- or disease-related narrow therapeutic window; altered clearance; barrier disorders.	Weight-based dosing that does not exceed 4.5 mg/kg plain lidocaine or 7 mg/kg lidocaine with epinephrine in children and non-frail adults undergoing infiltration anesthesia, with stricter limits of 4–5 mg/kg lidocaine in frail elderly patients or those with significant hepatic dysfunction; preference for dilute, buffered lidocaine–epinephrine solutions; avoidance of prilocaine- and benzocaine-containing products in infants; standard agents in immunocompromised or transplant recipients, with dose reductions based on hepatic and renal function [7,8,13,14]	Rigorous cumulative dose tracking; shorter topical exposure on diseased skin; lower threshold for monitoring; explicit counselling and consent addressing age-specific risks and warning signs.

## Keypoints

- Dilute epinephrine (1:100,000–1:200,000) with amide anesthetics is safe in well-perfused end-arterial sites (digits, nose, ear, penis) when combined with vascular risk stratification and ready access to phentolamine rescue.
- Barrier-compromised skin (burns, ulcers, inflamed dermatoses) acts as an “absorption amplifier,” making high-dose or occluded topicals a major driver of LAST and methemoglobinemia, especially in infants and frail elderly, so dose, area, and contact time must be sharply reduced.
- For large denuded or high-risk fields, dilute tumescent or field infiltration with lidocaine–epinephrine provides safer, more controllable pharmacokinetics than escalating topical anesthetic doses.
- Systemic toxicity risk is strongly influenced by cumulative dosing, comorbid hepatic/cardiac disease, age, and drug interactions, but severe LAST has become a largely manageable event with early recognition and lipid emulsion therapy.
- A context-sensitive framework integrating vascular status, barrier integrity, host pharmacokinetics, and office preparedness (including phentolamine and 20% lipid emulsion kits) enables safe local anesthesia in traditionally high-risk dermatologic scenarios.
